# Clotrimazole-Induced Oxidative Stress Triggers Novel Yeast Pkc1-Independent Cell Wall Integrity MAPK Pathway Circuitry

**DOI:** 10.3390/jof7080647

**Published:** 2021-08-09

**Authors:** Ángela Sellers-Moya, Marcos Nuévalos, María Molina, Humberto Martín

**Affiliations:** Departamento de Microbiología y Parasitología, Facultad de Farmacia, Instituto Ramón y Cajal de Investigaciones Sanitarias (IRYCIS), Universidad Complutense de Madrid, 28040 Madrid, Spain; angselle@ucm.es (Á.S.-M.); marnueva@ucm.es (M.N.)

**Keywords:** yeast, cell wall integrity, MAPK, phosphorylation, azoles, clotrimazole

## Abstract

Azoles are one of the most widely used drugs to treat fungal infections. To further understand the fungal response to azoles, we analyzed the MAPK circuitry of the model yeast *Saccharomyces cerevisiae* that operates under treatment with these antifungals. Imidazoles, and particularly clotrimazole, trigger deeper changes in MAPK phosphorylation than triazoles, involving a reduction in signaling through the mating pathway and the activation of the MAPKs Hog1 and Slt2 from the High-Osmolarity Glycerol (HOG) and the Cell Wall Integrity (CWI) pathways, respectively. Clotrimazole treatment leads to actin aggregation, mitochondrial alteration, and oxidative stress, which is essential not only for the activation of both MAPKs, but also for the appearance of a low-mobility form of Slt2 caused by additional phosphorylation to that occurring at the conserved TEY activation motif. Clotrimazole-induced ROS production and Slt2 phosphorylation are linked to Tpk3-mediated PKA activity. Resistance to clotrimazole depends on HOG and CWI-pathway-mediated stress responses. However, Pkc1 and other proteins acting upstream in the pathway are not critical for the activation of the Slt2 MAPK module, suggesting a novel rewiring of signaling through the CWI pathway. We further show that the strong impact of azole treatment on MAPK signaling is conserved in other yeast species.

## 1. Introduction

Signal transduction pathways mediated by mitogen-activated protein kinases (MAPKs) are essential for eukaryotic cells to respond to external stimuli, regulating vital cell processes such as proliferation, differentiation, survival, and apoptosis. As they play a central role in cellular homeostasis, compromised MAPK pathways may be involved in the development of serious diseases such as cancer or neurodegenerative disorders [1]. These pathways are composed of a module of three evolutionarily conserved kinases that are activated by sequential phosphorylation: a MAP kinase kinase kinase (MAPKKK), a MAP kinase kinase (MAPKK), and a MAP kinase (MAPK) [2]. For full activation, MAPKs need to be phosphorylated by MAPKKs on conserved T (threonine) and Y (tyrosine) within the activation domain [3]. Activated MAPKs then phosphorylate a diverse set of target proteins with different functions, including transcription factors, which are fundamental to the adaptive response.

In the yeast *Saccharomyces cerevisiae,* five MAPK routes govern mating, filamentous growth, osmolarity, spore wall assembly, and cell wall integrity [3]. The pheromone response pathway is crucial for yeast to initiate the process of mating differentiation [4]. Pheromones from the opposite mating partner are detected by G-protein-coupled receptors, transmitting the signal to the p21-activated kinase (PAK) Ste20 through G-protein activation. The MAPK module is composed of MAPKKK Ste11, MAPKK Ste7, and MAPK Fus3, which are attached to the scaffold protein Ste5. Kss1, the homolog MAPK of Fus3 responsible for filamentous and invasive growth, is also phosphorylated in response to pheromones but in a more transient manner than Fus3 [5]. The High-Osmolarity Glycerol (HOG) pathway is necessary for adaptation to hyperosmotic stress and is composed of two branches [6]. The first branch is named after the protein Sho1, a membrane-localized scaffold protein that interacts with the sensors Hkr1 and Msb2. The signal is transmitted to Ste20, which in turn activates MAPKKK Ste11. The second branch is named after the sensor histidine kinase Sln1, which signals through the phosphotransfer protein Ypd1 to the response regulator Ssk1, which activates the redundant MAPKKKs Ssk2 and Ssk22. Both branches converge in MAPKK Pbs2, leading to MAPK Hog1 activation [7].

The Cell Wall Integrity (CWI) pathway is necessary to maintain cell wall stability in response to cell surface stress, although it can also be activated by other stimuli not directly related to cell wall damage such as oxidative or genotoxic stress [8]. Perturbations in the cell wall or the plasma membrane are detected by a group of membrane-spanning sensors (the Wsc-type sensors Wsc1, Wsc2, and Wsc3, Mid2, and Mtl1) [9], which activate Rho1 GTPase through GDP/GTP exchange factors (GEFs) Rom2, Rom1, and Tus1 [10]. Activated Rho1 interacts with the protein kinase Pkc1, which in turn activates the MAPK module composed of MAPKKK Bck1, two redundant MAPKKs Mkk1 and Mkk2, and MAPK Slt2 [11]. Once activated, Slt2 can phosphorylate a diverse set of substrates, including the transcription factors Rlm1 and SBF complex (consisting of Swi4 and Swi6), which regulate the expression of genes implicated in cell-wall repair such as *MLP1* and *FKS1*, respectively [12]. In addition to Slt2 dual phosphorylation in its conserved TEY domain, other residues have been described to be phosphorylated under specific conditions. This is the case with caffeine treatment, which leads to DNA damage checkpoint kinases-mediated Slt2 phosphorylation at Ser423 and Ser428, affecting Slt2 interaction with Swi4 [13].

In recent years, the emergence of pathogenic fungi resistant to distinct antifungals has been alarming, as they have rapidly developed diverse resistance mechanisms against the different classes of drugs used in the clinic [14]. It is thus important to understand fungal cell responses to antifungal treatment, as this knowledge will aid the development of new therapeutic strategies to overcome this problem. Azoles are the most widely used class of antifungals. They are divided in two families, including imidazoles, which are commonly used for the treatment of superficial mycoses, and triazoles, which are preferably used in systemic mycosis due to their better pharmacokinetic profile, spectrum of activity, and safety [15]. Within imidazoles, clotrimazole is effective against skin, vulvovaginal, and oropharyngeal fungal infections and has become a drug of interest for the treatment of other diseases such as malaria and some cancers [16]. The main effect of azole antifungals is the inhibition of the ergosterol biosynthetic pathway, and thus alterations in the permeability and fluidity of the cell membrane [17]. Additionally, some of the azoles were reported to have a more complex mode of action, triggering other effects such as the induction of farnesol production [18] or the generation of reactive oxygen species (ROS) [19,20]. Regarding the effect of azoles on MAPK signaling, little is known. Activation of CWI, HOG, and Cek1 routes was observed in *Candida albicans* after exposure to the triazole fluconazole [21], and deletion of CWI pathway components encoding genes *PKC1*, *BCK1*, *SLT2,* and *SWI4* rendered cells of both *S. cerevisiae* and *C. albicans* more sensitive to fluconazole [22]. However, the effect of imidazoles on fungal MAPK pathways has not yet been explored.

The use of *S. cerevisiae* as a model system in antifungal research is currently considered opportune [23]. Here, we exploited the powerful genetics and biochemistry of this yeast to gain insight into the nature of the stress imposed by clotrimazole and the MAPK signaling circuitry that enables resistance to this azole.

## 2. Materials and Methods

### 2.1. Yeast Strains and Plasmids

The *Saccharomyces cerevisiae* strains used in this work were the wild-type BY4741 (*MAT*a *his3Δ1 leu2Δ0 met15Δ0 ura3Δ0*) and the isogenic *kanMX4* deletion mutants from Euroscarf (Frankfurt, Germany); the YMJ29 strain (BY4742 isogenic, *MATα his3Δ1 leu2Δ0 lys2Δ0 ura3Δ0 mkk2Δ::kanMX4 mkk1Δ::SpHIS5*) [24]; the wild-type CML128 (*MAT*a *leu2-3 112 ura3-52 trp1 his4 can1r*) and its isogenic *pkc1::LEU2* mutant strain MML344 [25]; WT-RLM1Myc (BY4741 isogenic *RLM1-6MYC::HIS3*) and *slt2Δ*-RLM1Myc (BY4741 isogenic *slt2Δ::kanMX4 RLM1-6MYC::HIS3*) [26]; mutant *rlm1Δ* (BY4741 isogenic *rlm1Δ::TRP1*) [27]; and mutant *bcy1Δ* (BY4741 isogenic *bcy1Δ::KanMX4*) [28]. Mutant *pkc1Δ* (BY4741 isogenic *pkc1Δ::HIS3*) was kindly provided by Dr. Javier Arroyo. Strains YSTH1 (Y3656 isogenic, *MATα can1∆::MFA1pr-HIS3-MFα1pr-LEU2 his3 leu2 ura3 met15 lys2 HO::natMX6::SSB1*) and YSTH2 (Y3656 isogenic, *HO::pMLP1-MKK1^S386P^-tADH-natMX6::SSB1*) including IPAC [29] were a gift from Dr. Elena Jiménez-Gutiérrez. The WT-SWI6Myc strain (BY4741 isogenic *SWI6-6MYC::LEU2*) was obtained by amplifying the pRS305m plasmid containing a Myc_6_ epitope [30] with primers 5′-CTGACGAAATGCAAGATTTTTTAAAAAAGCATGCTTCAGCTATGGAGCAAAAGCTCATTTCTG-3′ and 5′-CAAATAAAGTCATAAAAGTTAATGCAATGAAATCACATGCCCTTTTTAAGCAAGGATTTTC-3′ (regions homologous to the *SWI6* sequence are underlined) and integrating the PCR product into the *SWI6* locus. To obtain BY4741-T (BY4741 isogenic *trp1Δ::NatMX6*), the disruption cassette amplified with the primers 5′-GCACGTGAGTATACGTGATTAAGCACACAAAGGCAGCTTGGCGTACGCTGCAGGTCGAC-3′ and 5′-CAATACTTAAATAAATACTACTCAGTAATAACCTATTTCTTAGCATCGATGAATTCGAGCTCG-3′ (regions homologous to *TRP1* are underlined) was integrated into the *TRP1* locus.

For the experiments with different yeast species, the wild-type strains used were *Saccharomyces cerevisiae* BY4741, *Pichia pastoris* GS115 (Invitrogen, Waltham, MA, USA), *Pichia anomala* 1026, *Kluyveromyces lactis* 1049, *Hansenula mrakii* K9, *Candida glabrata* ATCC 2001 CBS138, *Meyerozyma guilliermondii* TP11010 CNM-CL9533, *Candida albicans* 4482A, and *Candida tropicalis* 4458 CNM-CL9537. Strains were kindly provided by Ana Alastruey-Izquierdo from the Instituto de Salud Carlos III, Spain (CNM-strains) and by Elvira Marín (Departamento de Microbiología y Parasitología, Universidad Complutense de Madrid, Spain).

The plasmids used in this work were pRS316 [31], pRS316-*SLT2* [32], p2313 (YEp351-*SLT2-FLAG*) [33], pVD67 (pGRU2-*PKC1-GFP*) [34], pRS314 [31], pRS314-RLM1-HA, pRS314-*rlm1*-3m-HA, pRS314-*rlm1*-L324A/V326A-HA, pRS314-*rlm1*-10m-HA [27,35], p*MLP1-LacZ* [36], and YEplac112-Ilv6-mCherry [37].

### 2.2. Culture Conditions

Depending on the experimental approaches used, yeast cells were grown on YPD (2% glucose, 2% peptone, and 1% yeast extract) or SD medium (a 0.17% yeast nitrogen base, 0.5% ammonium sulfate, and 2% glucose) supplemented with the required amino acids, either broth or agar. For routine cultures, yeast cells were grown overnight at 24 or 30 °C on YPD or SD medium. The cultures were refreshed to an OD_600_ of 0.3 and grown on YPD for 2 h at the same temperature to allow cells to enter into the exponential phase. For further experiments, the cultures were either grown under the same conditions (the untreated samples) or supplemented with the corresponding compound. The compounds tested were clotrimazole, ketoconazole, itraconazole, fluconazole, chlorpromazine, N-acetylcysteine (Sigma-Aldrich, St. Louis, MO, USA), and Congo red (Merck, Darmstadt, Germany). Cells were collected at the indicated times and processed as mentioned in the following assays. For the experiments with different yeast species, YPD was supplemented with 1 M MES buffer.

### 2.3. Multi-Well-Plate Sensitivity Assay

Yeast cells from an overnight culture were diluted to a final OD_600_ of 0.005 and cultured at 30 °C in multi-well plates containing YPD with serial dilutions of the azoles clotrimazole, ketoconazole, itraconazole, and fluconazole at concentrations from 0.0976 to 100 µg/mL, as well as a control without compound. Growth was determined as OD_595_ after 24 h of static incubation using a microplate reader (Bio rad 680; Bio rad, Hercules, CA, USA).

### 2.4. Yeast Drop Dilution Growth Assays

Growth assays on solid media were performed by culturing cells in YPD to an OD_600_ of 0.5 and spotting samples (5 µL) of 10-fold dilutions of the cell suspensions onto the surface of YPD plates, followed by incubation at 30 °C for 72 h. YPD plates were supplemented with 1 µg/mL of clotrimazole, 30 µg/mL of Congo red, 20 mM of N-acetylcysteine, or 1 M of sorbitol (PanReac AppliChem ITW Reagents, Barcelona, Spain) when indicated.

### 2.5. Preparation of Yeast Extracts and Immunoblotting Analysis

The preparation of yeast extracts from routine cultures, fractionation by SDS-PAGE, and transfer to nitrocellulose membranes were described previously [38]. When indicated, staining of membranes with 0.5% Ponceau in 5% acetic acid was performed. Rabbit monoclonal anti-phospho-p44/42 (Erk1/2, Thr202/Tyr204, #4370; Cell Signaling; Danvers, MA, USA), rabbit monoclonal anti-phospho-p38 (Thr180/Tyr182, #9215; Cell Signaling), mouse monoclonal anti-Mpk1 (E-9; Santa Cruz Biotechnology, Dallas, TX, USA), rabbit polyclonal anti-Hog1 (y215; Santa Cruz), mouse monoclonal anti-Myc clone 4A6 (Millipore, Burlington, MA, USA), mouse monoclonal anti-HA (12CA5, Sigma-Aldrich,), and rabbit polyclonal anti-G6PDH (Sigma-Aldrich) antibodies were used to recognize dually phosphorylated Slt2, Kss1, and Fus3; dually phosphorylated Hog1; Slt2; Hog1; Myc-tagged proteins; HA-tagged proteins; and G6PDH, respectively. Detection of Glucose-6-phosphate dehydrogenase (G6PDH) encoded by the gene *ZWF1* was used as a loading control. The primary antibodies were detected using a fluorescence-conjugated secondary antibody with an Odyssey Infrared Imaging System (Li-Cor Biosciences; Lincoln, NE, USA).

### 2.6. Alkaline Phosphatase Assay

Collected cells were resuspended in cold lysis buffer (50 mM TrisHCl at pH 7.5, 150 mM NaCl, 50 mM NaF, 5 mM sodium pyrophosphate, 50 mM β-glycerol-phosphate, 0.1% NP 40, and 10% glycerol) supplemented with 1 mM phenylmethylsulfonyl fluoride (PMSF) and a protease inhibitors mixture (Thermo Scientific, Waltham, MA, USA). Cells were lysed using glass beads, and the protein concentration of the extracts was measured at 280 nm and normalized with lysis buffer. For immunoprecipitation, 200 µL of extracts were incubated with 40 µL of FLAG-Sepharose beads (Sigma-Aldrich) overnight at 4 °C. Beads were extensively washed with alkaline phosphatase buffer (50 mM Tris-HCl at pH 7.5, 100 mM NaCl, 10 mM MgCl_2_, and 1 mM DTT, adjusted to a pH of 7.9) and finally resuspended with 400 µL of the same buffer. When indicated, 15 µL of alkaline phosphatase from calf intestine (ALP) or 40 µL of 100 mM sodium orthovanadate (Na_3_VO_4_), as a phosphatase inhibitor, to a final concentration of 10 mM were added. Samples were incubated at 37 °C for 1 h and centrifugated at 3000 rpm for 1 min. We added 2× SDS loading buffer. Proteins were boiled for 5 min and then analyzed by SDS-PAGE and immunoblotting.

### 2.7. Microscopy Techniques

For mCherry in vivo fluorescence microscopy, yeast transformants were cultured as usual and cells were collected by centrifugation of 1 mL of culture at 2500 rpm. For actin staining, cells were cultured as usual, fixed with *p*-formaldehyde, and stained with rhodamine–phalloidin as described previously [39]. Samples were prepared for visualization and the microscope used was an Eclipse TE2000U (Nikon, Tokyo, Japan) with the appropriate sets of filters. Digital images were acquired with an Orca C4742-95-12ER charge-coupled device camera (Hamamatsu, Hamamatsu city, Japan) and processed with HCImage software (Hamamatsu).

### 2.8. Quantitative RT-PCR Assays

RNA isolation and purification from yeast cells were conducted using the NucleoSpin RNA Mini kit for RNA purification (Macherey-Nagel, Düren, Germany), following the manufacturer’s instructions. Real-time quantitative PCR (RT-qPCR) assays were performed as previously detailed [40]. For quantification, the abundance of each gene was determined relative to the standard transcript of *ACT1,* and the final data of relative gene expression were calculated following the 2^−2ΔΔCt^ method [41]. Primers were kindly provided by Dr. Javier Arroyo.

### 2.9. Flow Cytometry Evaluation of Oxidative Damage

For the detection of oxygen free radicals (ROS) and variations in the mitochondrial membrane potential, yeast cells were grown on YPD and cultured as usual, and 2.5 µg/mL of dihydroethidium (DHE) or 5 µg/mL of rhodamine 123, respectively, were added to 1 mL of each sample and incubated at 24 °C for 5 and 30 min. Then, samples were diluted 1:10 in PBS and analyzed on a FACScalibur flow cytometer (Becton Dickinson, Franklin Lakes, NJ, USA). Data were analyzed with FlowJo software (Becton Dickinson).

## 3. Results

### 3.1. Clotrimazole Activates the CWI and HOG Pathways and Attenuates the Mating Pathway

**Figure 1:**
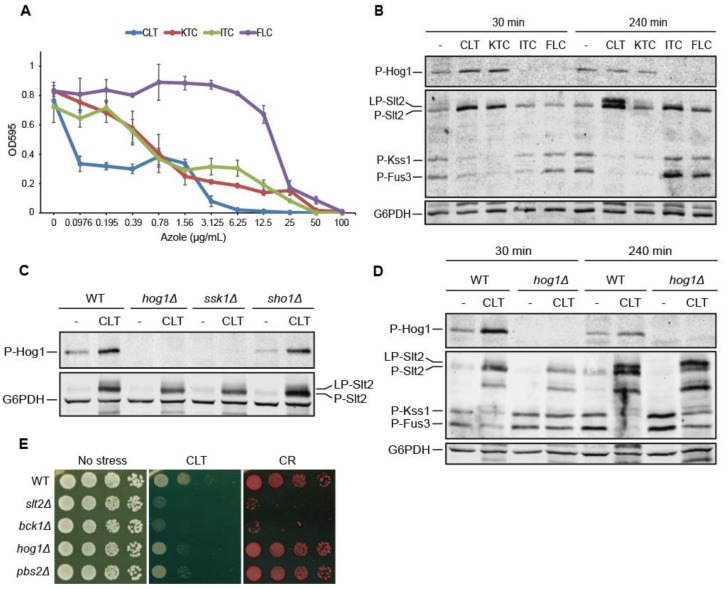
Impact of azole treatment on *S. cerevisiae* MAPK signaling. (**A**) Multi-well-plate sensitivity assay of the BY4741 strain for the indicated concentrations of clotrimazole (CLT), ketoconazole (KTC), itraconazole (ITC), and fluconazole (FLC). Cells were cultured at 30 °C for 24 h and the optical density at 595 nm (OD_595_) was measured. Data are presented as the mean of three independent experiments. Error bars indicate standard deviation. (**B**) Western blotting analysis of extracts of BY4741 cells treated with 50 µg/mL of CLT; 400 µg/mL of KTC, ITC, and FLC; or without any azole (-) for the indicated times at 24 °C. Dually phosphorylated Hog1 was detected with anti-phospho-p38; dually phosphorylated Slt2, Kss1, and Fus3 were detected with anti-phospho-p44/42; and G6PDH, encoded by the gene *ZWF1* (as the loading control), with anti-G6PDH. (**C**,**D**) Western blotting analysis of cell extracts of BY4741 (WT) and the indicated isogenic mutant strains. Cells were cultured without (-) or with 50 µg/mL of CLT for 30 min (**C**) or 30 and 240 min (**D**) at 24 °C. Dually phosphorylated MAPKs and G6PDH were detected as described in (**B**). Representative blots from three independent experiments are shown. (**E**) Drop dilution growth assay of BY4741 (WT) and the indicated isogenic mutant strains. Ten-fold serial dilutions of cell suspensions were spotted onto YPD plates in the absence (no stress) or presence of 1 µg/mL of CLT or 30 µg/mL of Congo red (CR) and incubated at 30 °C for 72 h. A representative assay from three independent experiments is shown.

To test the changes in yeast MAPK circuitry imposed by antifungal azoles, we began by analyzing the sensitivity of the reference *S. cerevisiae* wild-type strain (BY4741) to four clinically relevant azoles: two imidazoles, clotrimazole (CLT) and ketoconazole (KTC); and two triazoles, itraconazole (ITC) and fluconazole (FLC). As observed in Figure 1A, CLT was the most potent azole, exhibiting inhibitory concentrations eight-fold lower than the rest of the azoles, with the sensitivity to FLC being the lowest.

We thus analyzed the impact of treating exponentially growing yeast cells with an inhibitory CLT concentration of 50 µg/mL and a comparable eight-fold-higher concentration of KTC, ITC, and FLC at two different time points on MAPK phosphorylation (Figure 1B). Treatment with both imidazoles, CLT and KTC, led to the activation of the MAPKs Hog1 and Slt2 of the HOG and CWI pathways by dual phosphorylation, respectively, after a short exposure of 30 min. Interestingly, at a longer exposure time of 240 min, in addition to the increase in Slt2 phosphorylation levels, a slower phospho-Slt2 migrating band was apparent after treatment with either CLT or KTC. The phosphorylated Slt2 form that ran with low mobility is hereafter called lowphospho-Slt2 (LP-Slt2), in contrast with the canonical phospho-Slt2 (P-Slt2). The stimulation of yeast cells with either of these two imidazole drugs induced a reduction in the basal phosphorylation levels of the MAPKs from the pheromone response pathway Fus3 and Kss1. After the longest exposure time, triazoles prompted some activation of Slt2, but neither the appearance of the LP-Slt2 form, nor the activation of Hog1, nor a reduction in Fus3 and Kss1 phosphorylation occurred. All these results indicate that imidazoles, but not triazoles, elicit specific and profound changes in yeast-MAPK-mediated signal transduction. Since CLT imposed the most severe changes in MAPK phosphorylation, we decided to focus on the effect of this imidazole on signaling in yeast cells.

We first identified the HOG pathway branch that senses and transmits the stimulus in response to CLT. In contrast to Sho1, removal of Ssk1 impaired signaling to Hog1 (Figure 1C), indicating that CLT is detected and transmitted through the branch of the HOG pathway in which the Sln1-Ypd1-Ssk1 phospho-relay system operates. We also explored the crosstalk between HOG and other MAPK pathways, as the HOG pathway regulates signaling through the CWI and mating pathways under some stimuli [29,42,43]. Lack of this MAPK caused only a slight reduction in Slt2 phosphorylation at 30 min without interfering with the appearance of the LP-Slt2 form, indicating that Slt2 activation does not depend on this crosstalk mechanism (Figure 1D). In contrast, CLT-imposed Fus3 and Kss1 dephosphorylation disappeared at 30 min and was partially impaired at 240 min in the absence of Hog1, suggesting that Hog1 modulates signaling through the mating pathway in response to this azole.

The importance of the essential components for signaling through the HOG and CWI MAPK pathways for resistance to CLT was also analyzed. As observed in Figure 1E, *hog1Δ* and *pbs2Δ* mutants exhibited a slight sensitivity to CLT, whereas *slt2Δ* and *bck1Δ* mutants displayed a significantly higher sensitivity to the antifungal drug. In contrast, only the CWI pathway kinases proved to be necessary for cells to grow under the cell-wall-altering agent Congo red (CR). These results confirm the relevance of these pathways for responding to this azole.

### 3.2. Clotrimazole Induces a Phosphorylation-Dependent Mobility Shift in Slt2

**Figure 2:**
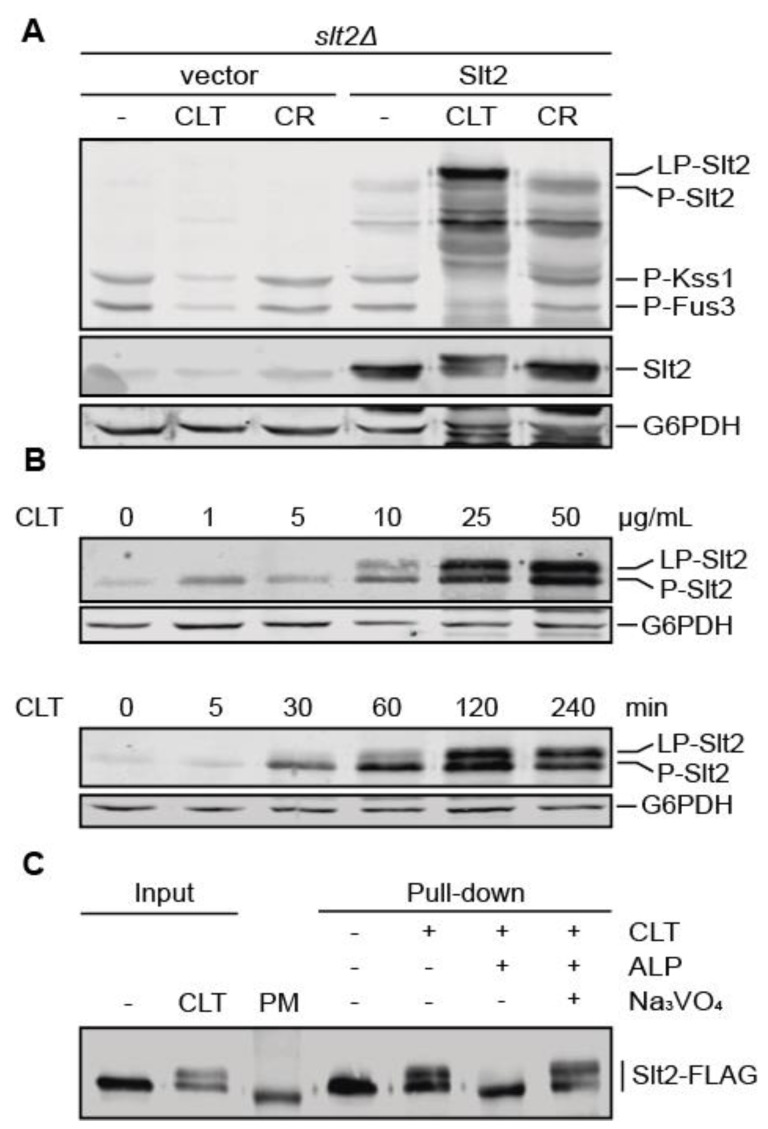
Characterization of Slt2 phosphorylation and LP-Slt2 emergence upon clotrimazole treatment. (**A**) Western blotting analysis of extracts of BY4741 *slt2Δ* cells transformed with either the empty vector pRS316 or pRS316-*SLT2* and cultured with 50 µg/mL of CLT, 30 µg/mL of CR, or without treatment (−) for 4 h at 24 °C. Dually phosphorylated Slt2, Kss1, and Fus3; Slt2 protein; and G6PDH (as a loading control) were detected with anti-phospho-p44/42, anti-Mpk1, and anti-G6PDH, respectively. (**B**) Western blotting analysis of extracts of BY4741 cells treated with increasing concentrations of CLT for 4 h (upper panel) and with 50 µg/mL of CLT for different times (lower panel) at 24 °C. Dually phosphorylated Slt2 and G6PDH were detected as described in (**A**). (**C**) Alkaline phosphatase assay of BY4741 *slt2Δ* cells transformed with p2313 plasmid (Slt2-FLAG) and cultured with (+) or without (−) 50 µg/mL of CLT for 4 h at 24 °C. Immunoprecipitated Slt2-FLAG with anti-FLAG antibodies (pull-down) was treated with (+) or without (−) alkaline phosphatase (ALP) from calf intestine in the absence (−) or presence (+) of sodium orthovanadate (Na_3_VO_4_), a phosphatase inhibitor. Cell extracts (input) were also analyzed. Slt2-FLAG was detected with anti-Mpk1 antibody. PM corresponds to the lane loaded with the molecular weight protein marker. Representative blots from three (**A**,**B**) and two (**C**) independent experiments are shown.

To further investigate the effect of CLT on MAPK signaling, we compared Slt2, Fus3, and Kss1 phosphorylation under CLT and CR treatment in an *slt2Δ* strain transformed with either a centromeric plasmid carrying the *SLT2* gene or the corresponding empty vector (Figure 2A). Whereas CLT led to the appearance of LP-Slt2 and stronger Slt2 phosphorylation than CR, neither the absence nor the presence of this MAPK had an impact on reducing the amount of phosphorylated Fus3 and Kss1 upon treatment with this azole. Interestingly, the use of anti-Slt2 antibodies showed that a significant proportion of the overall amount of Slt2 within the cell experienced the SDS-PAGE mobility shift.

Having found that Slt2 shifted upward when cells were treated with imidazoles, we next sought to determine the dose-dependence and kinetics of this Slt2 modification upon CLT treatment. As shown in Figure 2B, Slt2 activation was dependent on CLT concentration and exposure time. Thus, treatment with CLT concentrations higher than 10 µg/mL and exposure times longer than 30 min resulted in a strong and progressive increase in both Slt2 phosphorylation and LP-Slt2 levels.

To gain insight into the origin of this LP-Slt2 emergence with CLT treatment, we assessed whether it can be promoted by phosphorylation events. Therefore, we analyzed the effect of phosphatase treatment on Slt2 electrophoretic mobility (Figure 2C). The removal of phosphates from Slt2 purified from CLT-treated cells led to the disappearance of the low-mobility form, suggesting that Slt2 can be phosphorylated in one or more sites additional to the conserved T and Y at the activation loop in response to CLT stimulation.

### 3.3. Pkc1 Is Not Critical for Clotrimazole-Induced Signal Transduction through the CWI Pathway

To characterize how the CWI pathway senses the stimulus and transmits the signal when cells are exposed to CLT, we studied the contribution of different components of this pathway. CR-treated cells were analyzed in parallel, since the proteins involved in signal transduction for this stimulus are well-known [44,45].

**Figure 3:**
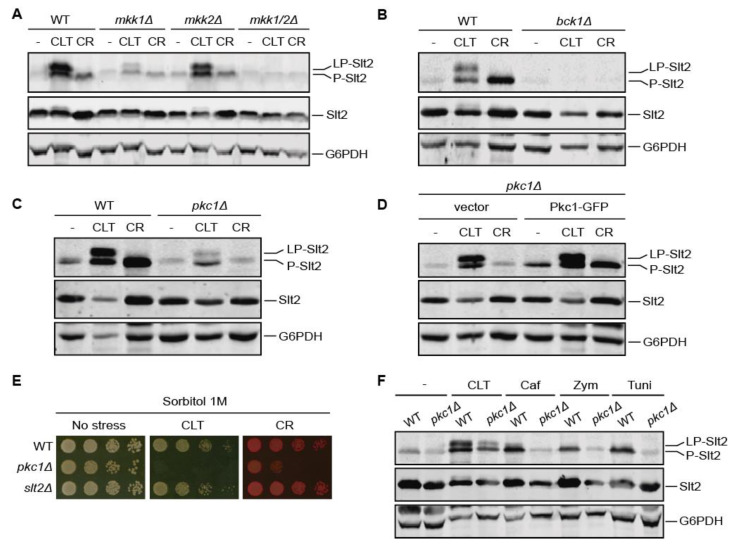
Involvement of protein kinases operating at the CWI pathway in clotrimazole-induced signaling to Slt2. (**A**–**C**) Western blotting analysis of extracts of BY4741 (WT) and the indicated isogenic mutant strains. Cells were cultured without stimulus (-), with 50 µg/mL of CLT, or with 30 µg/mL of CR for 4 h at 24 °C. Dually phosphorylated Slt2, Slt2 protein, and G6PDH (as a loading control) were detected with anti-phospho-p44/42, anti-Mpk1, and anti-G6PDH, respectively. (**D**) Western blotting analysis of *pkc1∆* cells transformed with either the empty vector pRS316 or with pVD67 (Pkc1-GFP). Cells were cultured and proteins were detected as described in (**A**–**C**). (**E**) Sensitivity of BY4741 (WT) and the indicated mutant strains to CLT and CR per drop dilution growth assay. Ten-fold serial dilutions of cell suspensions were spotted onto YPD plates supplemented with 1 M sorbitol in the absence (no stress) or presence of 1 µg/mL of CLT or 30 µg/mL of CR, and incubated at 30 °C for 72 h. A representative assay from three independent experiments is shown. (**F**) Western blotting analysis of extracts of BY4741 (WT) and its isogenic *pkc1Δ* strain. Cells were cultured without stimulus (-), with 50 µg/mL of CLT, 8 mM of caffeine (Caf), 0.4 U/mL of zymolyase (Zym), or 5 µg/mL of tunicamycin (Tuni) for 2 h at 24 °C. Proteins were detected as described in (**A**–**C**). Representative blots from two independent experiments are shown.

As observed for CR, CLT-induced Slt2 phosphorylation was abolished in the absence of both Mkk1 and Mkk2 or Bck1, indicating the essential role of protein kinases from the MAPK module in conveying signaling through the pathway (Figure 3A,B). As expected [46], Mkk1 played a central role in phosphorylating Slt2, but the low level of phosphorylated Slt2 observed in the *mkk1Δ* mutant was also accompanied by the appearance of a slow migrating band. This result reflects that this additional post-translational modification does not depend on the level of Slt2 activation.

Strikingly, and in contrast to what occurs with other CWI pathway stimuli such as CR, Pkc1 proved to be dispensable for CLT-induced Slt2 phosphorylation. As observed in Figure 3C, signaling through the CWI pathway in response to CLT was possible in the absence of Pkc1, leading to an increase in Slt2 phosphorylation, although P-Slt2 and LP-Slt2 levels were lower than in the wild-type strain. Therefore, when *pkc1∆* cells were transformed with a plasmid bearing Pkc1-GFP, the phosphorylation levels of Slt2 increased in CLT-treated cells and CR-induced activation of Slt2 was restored (Figure 3D). Drop growth assays in the presence of sorbitol were also performed (Figure 3E). The *pkc1∆* mutant, in contrast to the *slt2∆* one, was sensitive to CLT even in the presence of an osmotic stabilizer, suggesting a role for Pkc1 in cell defense against this antifungal, in addition to its role in CWI pathway signal transmission. As this was the first time that Pkc1 did not seem to be essential for CWI pathway signaling, we wanted to confirm these results. We thus performed the same experiments mentioned above using a *pkc1Δ* mutant derived from another yeast strain with a different genetic background and observed similar results (Figure S1).

To further ascertain the specificity of these observations for CLT treatment and not for other stimuli of the pathway, we also analyzed the activation of Slt2 in the *pkc1∆* mutant in response to caffeine, zymolyase, and tunicamycin. As shown in Figure 3F, none of these stimuli led to Slt2 activation in the absence of Pkc1, whereas CLT clearly prompted Slt2 phosphorylation to some extent. Altogether, these results show that, although Pkc1 seems to be important for the cell to respond to and survive CLT stress, an alternative route independent of Pkc1 is also capable of activating the MAPK module of the CWI pathway in response to this azole.

### 3.4. The Mechanosensor Wsc1 Is Involved in CWI Signaling Induced by Clotrimazole

**Figure 4:**
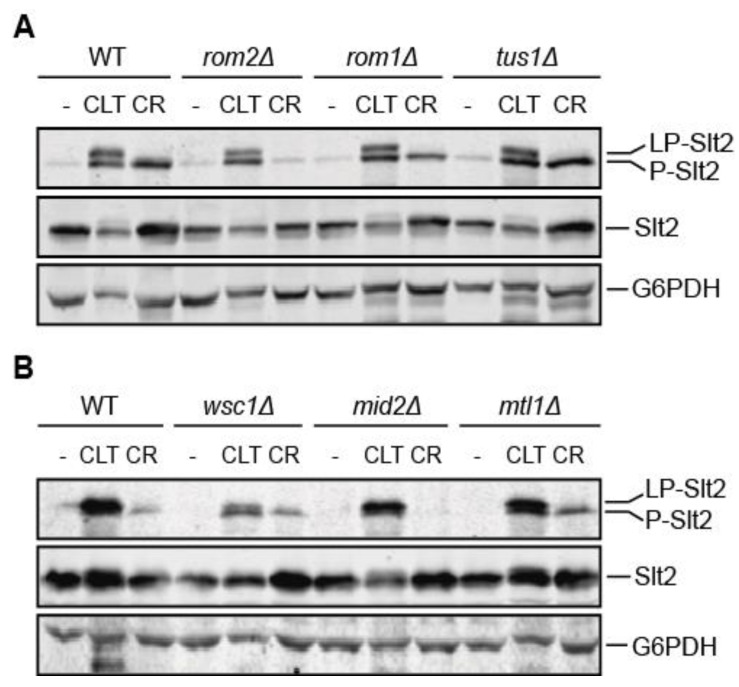
Involvement of components upstream of Pkc1 in clotrimazole-triggered signaling through the CWI pathway. (**A**,**B**) Western blotting analysis of extracts of BY4741 (WT) and the indicated isogenic mutant strains. Cells were cultured without stimulus (-), with 50 µg/mL of CLT, or with 30 µg/mL of CR for 4 h at 24 °C. Dually phosphorylated Slt2, Slt2 protein, and G6PDH (as a loading control) were detected with anti-phospho-p44/42, anti-Mpk1, and anti-G6PDH, respectively. Representative blots from three independent experiments are shown.

Since Pkc1 is not essential for CLT-induced Slt2 activation, we analyzed the importance of the CWI pathway components upstream of Pkc1 for signal transmission. Removal of any of the Rho1 GEFs, namely Rom1, Rom2, or Tus1, neither prevented Slt2 activation nor Slt2 band shift (Figure 4A). Notably, the effect of *ROM2* deletion on CR-induced Slt2 activation is in sharp contrast to that of CLT-induced Slt2 activation. Since Rom2 is likely the main GEF for Rho1 activation after cell-wall damage [45], this shows that CLT does not impact yeast cells as a typical cell-wall-stress stimulus.

The analysis of cells lacking each of the three main cell-wall-stress sensors (Wsc1, Mid2, and Mtl1) revealed that unlike the observation for Mid2 and Mtl1, the lack of Wsc1 considerably reduced Slt2 activation (Figure 4B), but without completely compromising MAPK activation. These results indicate an active but partial role of this mechanosensor in the response to CLT.

### 3.5. The Transcriptional Rlm1-Driven CWI Pathway Response to Clotrimazole Is Weak

**Figure 5:**
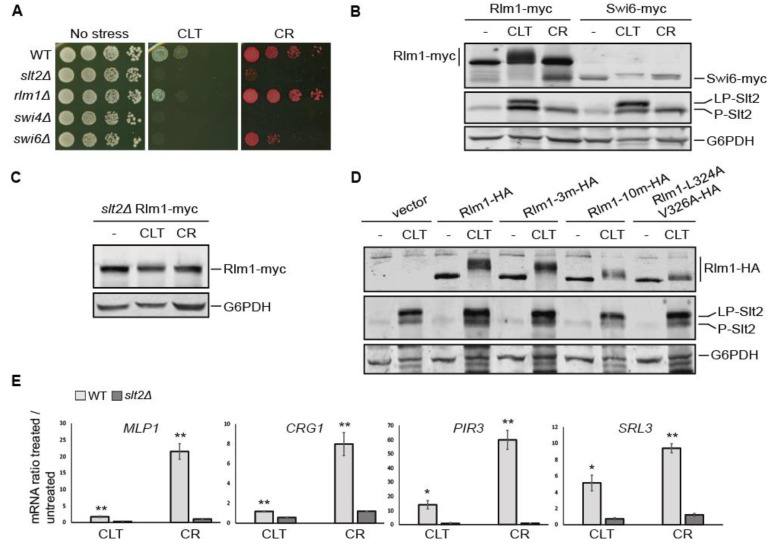
Analysis of the role of transcription factors Rlm1 and SBF in the clotrimazole response. (**A**) Drop dilution growth assay of BY4741 (WT) and the indicated isogenic mutant strains. Ten-fold serial dilutions of cell suspensions were spotted onto YPD plates in the absence (no stress) or presence of 1 µg/mL of CLT or 30 µg/mL of CR and incubated at 30 °C for 72 h. A representative assay from three independent experiments is shown. (**B**,**C**) Western blotting analysis of the extracts of WT-RLM1Myc, WT-SWI6Myc, and *slt2Δ*-RLM1Myc cells after treatment with 50 µg/mL of CLT, 30 µg/mL of CR, or without treatment (-) for 4 h at 24 °C. Rlm1-myc and Swi6-myc, dually phosphorylated Slt2, and G6PDH (as a loading control) were detected with anti-myc, anti-phospho-p44/42, and anti-G6PDH, respectively. (**D**) Western blotting analysis of extracts of the *rlm1∆* mutant strain transformed with the pRS314 plasmid bearing the indicated versions of Rlm1-HA. Cells were cultured without stimulus (-) or with 50 µg/mL of CLT for 4 h at 24 °C. Rlm1-HA was detected with anti-HA antibody and dually phosphorylated Slt2 and G6PDH were detected as described in (**A**). Representative blots from two independent experiments are shown. (**E**) Transcriptional expression of *MLP1*, *CRG1*, *PIR3*, and *SRL3* was analyzed by RT-qPCR in BY4741 WT and isogenic *slt2∆* strains after treatment with 50 µg/mL of CLT, 30 µg/mL of CR, or in basal conditions for 4 h at 24 °C. Values represent the expression ratio between treated and non-treated cells. Data correspond to the mean and standard deviation of three independent experiments. Student’s t-test was calculated between the WT and *slt2Δ* strains. * *p*-value < 0.05; ** *p*-value < 0.01.

To explore the transcriptional response to CLT driven by the CWI pathway, we studied the involvement of the principal transcription factors activated by Slt2, namely Rlm1 and the SBF complex (composed of Swi4 and Swi6 proteins). First, we analyzed the sensitivity of the mutants *rlm1Δ*, *swi4Δ*, and *swi6Δ* to CLT (Figure 5A). As in the case of CR, cells deficient in Swi4 and Swi6 showed a high sensitivity to the azole, similar to that observed for *slt2Δ* cells, whereas the lack of Rlm1 did not seem to severely impact CLT sensitivity. 

As shown in Figure 5B, both Rlm1 and Swi6 experienced Slt2-dependent phosphorylation after CLT exposure, which was detectable by a characteristic SDS-PAGE mobility shift [47,48]. Intriguingly, whereas the CLT-induced mobility shift in Swi6 followed the same pattern as that generated by CR, Rlm1 forms of slower mobility than those observed in the presence of CR appeared when cells were treated with this azole. This result suggests that under CLT treatment, Rlm1 is likely phosphorylated at sites additional to those targeted by other stimuli of the pathway. Moreover, this additional modification is dependent on the presence of Slt2 (Figure 5C). We thus used Rlm1 mutants in three and ten potential targets of Slt2-phosphorylation (Rlm1-3m-HA and Rlm1-10m-HA), which were previously described to be important for full Rlm1 activation [27], and analyzed their mobility shift after CLT treatment (Figure 5D). Both Rlm1 mutant versions migrated faster than the wild-type form, indicating that these phosphorylation sites are involved in a CLT-dependent mobility pattern. An Rlm1 version unable to interact with Slt2 (Rlm1-L3124AV326A-HA) [35] showed an even more reduced CLT-induced mobility shift. Note that the mobility shift was not completely abolished in either the Rlm1-10m-HA version or the Rlm1-L3124AV326A-HA version, again suggesting the presence of post-translational modifications in other residues apart from those already known for this transcription factor.

Surprisingly, when the expression of different Rlm1-dependent genes (*MLP1*, *CRG1*, *PIR3*, and *SRL3*) was analyzed by RT-qPCR (Figure 5E), the relative mRNA levels were notably lower in cells treated with CLT compared with those stimulated with CR, indicating that the observed additional phosphorylation of Rlm1 does not result in higher activity of this transcription factor. Nevertheless, while CLT-induced gene expression occurred at a low level, it showed a statistically significant reduction in the absence of Slt2, reflecting that this response is fully dependent on the CWI MAPK. To determine whether that induction was sufficient to generate a response of the pathway in cells exposed to this azole, we performed a sensitivity assay of cells bearing the *MLP1*-mediated positive feedback circuit called Integrity Pathway Activation Circuit (IPAC), which amplifies the signal and renders cells hypersensitive to CWI activators [29]. As confirmed in Figure S2, cells carrying the IPAC were considerably more sensitive to CLT than those without the circuit. This indicated that the minimal induction of the Rlm1-dependent *MLP1* promoter was sufficient to activate the circuitry and amplify the CLT-induced signal.

### 3.6. Oxidative Stress Is Necessary for Slt2 Activation by Clotrimazole

**Figure 6:**
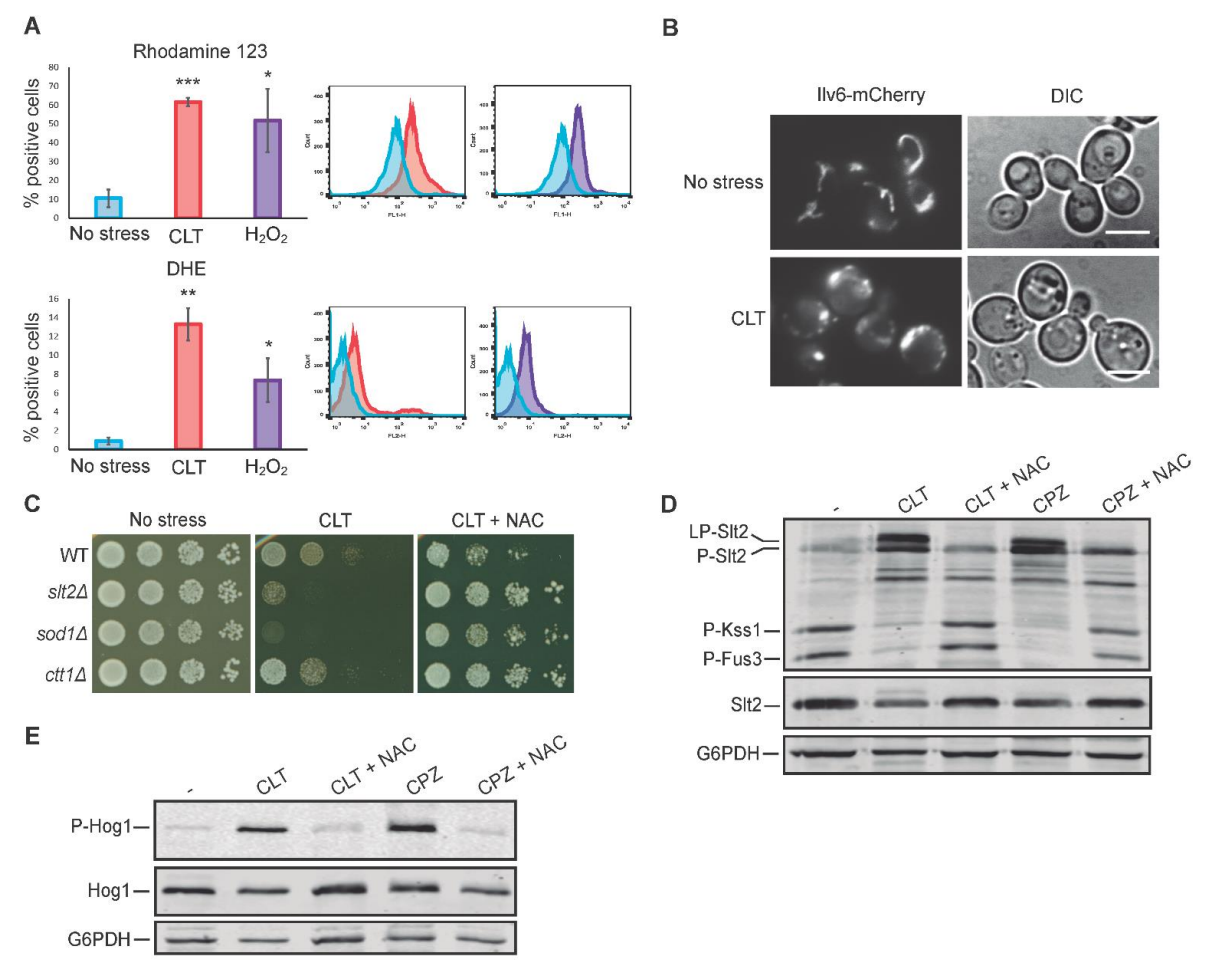
Effect of clotrimazole on mitochondrial morphology and oxidative stress: impact on MAPK phosphorylation. (**A**) Flow cytometry assay of the BY4741 strain after treatment with 50 µg/mL of CLT, 10 mM of H_2_O_2_, or basal conditions (no stress) for 4 h. Graphs represent the percentage of positive cells detected with flow cytometry that express a fluorescence signal for rhodamine 123 or dihydroethidium (DHE) fluorochromes. Data are presented as the mean of three independent experiments, and error bars indicate the standard deviation. Histograms from a representative experiment are shown. Student’s t-test was calculated between treated (CLT or H_2_O_2_) and non-treated (no stress) cells. *p*-value of < 0.05 (*), < 0.01 (**), or < 0.001(***). (**B**) Mitochondrial morphology alteration upon CLT treatment. Fluorescence and DIC microscopy images of BY4741-T cells transformed with YEplac112-Ilv6-mCherry plasmid after treatment with 50 µg/mL of CLT or in basal conditions (no stress) for 4 h. Representative photographs from two independent experiments are shown. Scale bar = 5 µm. (**C**) Sensitivity of BY4741 (WT) and the indicated isogenic mutant strains to CLT per a drop dilution growth assay. Ten-fold serial dilutions of cell suspensions were spotted onto YPD plates in the absence (no stress) or presence of 1 µg/mL of CLT and incubated at 30 °C for 72 h. Plates were supplemented with 20 mM of N-acetylcysteine (NAC) when indicated. A representative assay from three independent experiments is shown. (**D**,**E**) Western blotting analysis of the extracts of BY4741 cells treated with 50 µg/mL of CLT or 250 µM of chlorpromazine (CPZ) in combination with 20 mM of NAC, when indicated. Cells were cultured for 4 h (**D**) or 30 min (**E**) at 24 °C. Dually phosphorylated Slt2, Kss1, and Fus3; Slt2 protein; dually phosphorylated Hog1; Hog1 protein; and G6PDH (as a loading control) were detected with anti-phospho-p44/42, anti-Mpk1, anti-phospho-p38, anti-Hog1, and anti-G6PDH, respectively. Representative blots from three (**D**) and two (**E**) independent experiments are shown.

Since some azoles induce the accumulation of reactive oxygen species (ROS) in fungal cells [19,20], we studied whether CLT would induce oxidative stress and, if so, whether the produced ROS are responsible for CWI pathway activation and LP-Slt2 formation. To this end, we measured the mitochondrial membrane potential and ROS generation following CLT treatment by flow cytometry using the fluorescent dyes rhodamine 123 and dihydroethidium (DHE), respectively [49,50]. As a positive control, we treated cells with H_2_O_2_, a strong inducer of hydroxyl radical formation. After 4 h, we found that both CLT and H_2_O_2_ led to a significant increase in the fluorescence signal with both dyes (Figure 6A), confirming that CLT induces the formation of ROS and the alteration of mitochondrial activity. Furthermore, under CLT-treatment conditions, the mitochondrial morphology was clearly affected (Figure 6B). The branched tubular structure found in normal cells disappeared and deformed structures emerged, as revealed by analyzing the localization of the mitochondrial protein Ilv6 tagged with mCherry used as a mitochondrial marker.

To associate oxidative stress with CWI signaling following CLT stimulation, we used the antioxidant N-acetylcysteine (NAC). As shown in Figure 6C, the sensitivity of the *slt2∆* mutant to CLT was suppressed by NAC addition to the medium, suggesting that oxidative stress may be responsible for CWI pathway activation by CLT. The sensitivity to CLT displayed by cells deficient in antioxidant systems such as superoxide dismutase (Sod1) or catalase (Ctt1) was also eliminated by NAC, further supporting the oxidative effect of CLT and the utility of NAC in preventing it. The growth of all these deletion mutants was slightly higher than that of the WT strain onto CLT+NAC plates (Figure 6C), suggesting that these mutant strains might have some additional compensatory mechanisms to promote growth under oxidative stress that keep operating when this stress disappears. Additionally, we found that simultaneously exposing exponentially growing yeast cells to NAC and CLT significantly reduced Slt2 activation and eliminated the characteristic CLT-induced Slt2 shift (Figure 6D). The reductions in P-Kss1 and P-Fus3 basal levels induced by CLT were also abolished by NAC.

As we have observed for CLT, chlorpromazine (CPZ) has been reported to alter the plasma membrane, induce ROS production, and activate the yeast CWI pathway [51,52]. Therefore, we analyzed whether CPZ has a similar impact on yeast MAPK signaling to that of CLT. As shown in Figure 6D, this agent also provoked the appearance of LP-Slt2 and the reductions in P-Kss1 and P-Fus3 levels. Moreover, both effects were prevented by the addition of NAC. Hog1 phosphorylation was also induced by CPZ, and NAC hindered both the CPZ- and CLT-induced Hog1 activation (Figure 6E). These results suggest that the alteration in the plasma membrane together with the oxidative damage generated by any of these two compounds trigger this characteristic MAPK signaling response, not observed for other stimuli.

### 3.7. Tpk3-Mediated PKA Activity Modulates Slt2 Phosphorylation in Response to Clotrimazole 

**Figure 7:**
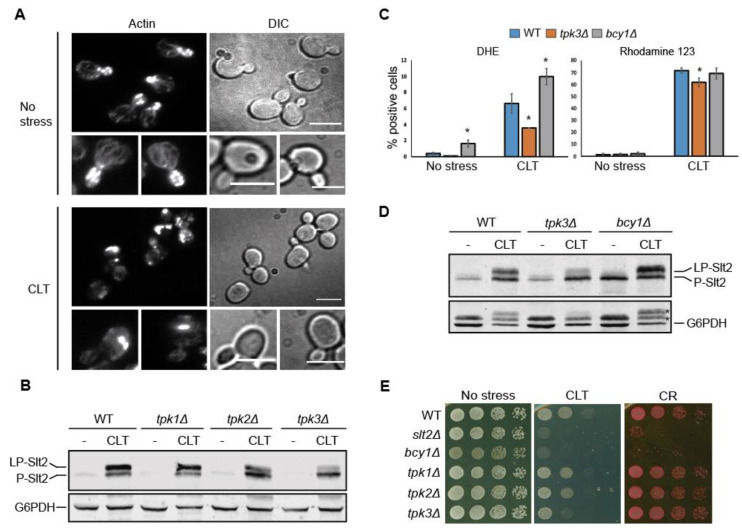
Involvement of the PKA kinase activity in clotrimazole-induced ROS production and Slt2 phosphorylation. (**A**) Rhodamine–phalloidin-based actin staining of BY4741 cells treated with 50 µg/mL of CLT or in basal conditions (no stress) for 4 h. Representative fluorescence and DIC photographs from three independent experiments are shown. Scale bar = 5 µm. (**B**) Western blotting analysis of the BY4741 (WT) strain and the indicated isogenic mutants. Cells were cultured without stimulus (-) or with 50 µg/mL of CLT for 4 h at 24 °C. Dually phosphorylated Slt2 and G6PDH (as a loading control) were detected with anti-phospho-p44/42 and anti-G6PDH, respectively. A representative blot from two independent experiments is shown. (**C**) Flow cytometry assay of the BY4741 (WT) strain and the isogenic *tpk3Δ* and *byc1Δ* mutants. Cells were either left untreated (no stress) or treated with 50 µg/mL of CLT for 4 h at 24 °C. Graphs represent the percentage of positive cells detected with flow cytometry that express a fluorescence signal for rhodamine 123 or dihydroethidium (DHE) fluorochromes. Data are presented as the mean of three independent experiments. Error bars indicate the standard deviation. Student’s t-test was calculated between the WT and mutant strains. * *p*-value < 0.05. (**D**) Western blotting analysis of the same samples as in (**C**). Proteins were detected as in (**B**). Asterisks in the lower panel showing G6PDH levels indicate bands that correspond to P-Slt2 and LP-Slt2. (**E**) Sensitivity of BY4741 *slt2Δ* and the same strains as in (**B,D**) to CLT and CR per the drop dilution growth assay. Ten-fold serial dilutions of cell suspensions were spotted onto YPD plates in the absence (no stress) or presence of 1 µg/mL of CLT or 30 µg/mL of CR and incubated at 30 °C for 72 h. A representative assay from two independent experiments is shown.

A strong correlation exists between ROS levels and the state of the actin cytoskeleton in yeast [53]. Additionally, miconazole was shown to induce yeast actin aggregation, which precedes ROS accumulation [54]. CLT-treated cells also displayed aberrant actin aggregates, as revealed by the actin dye rhodamine–phalloidin (Figure 7A), suggesting that actin alteration can contribute to CWI pathway activation.

Since actin aggregation was linked to Ras/PKA signaling [55], and the Ras/PKA pathway was related to the induction of mitochondrial ROS in response to distinct antifungal drug treatments [56], we next analyzed the involvement of this signaling pathway in Slt2 activation by CLT. First, we assessed the possible implication of each of the three PKA catalytic subunits, Tpk1, Tpk2, and Tpk3, on CWI signaling, as each of them can play specific functional roles [57,58]. As shown in Figure 7B, Tpk3 seemed to be partially involved in LP-Slt2 formation in response to CLT. Next, we measured the ROS levels and mitochondrial membrane potential of *tpk3∆* cells in parallel to cells deficient in Bcy1, the negative regulatory subunit of the PKA kinase (Figure 7C). Compared with wild-type cells, the absence of Tpk3 led to a significant reduction in ROS levels, whereas the lack of Bcy1 resulted in even higher ROS generation after CLT exposure, as measured using DHE fluorescent dye. Nevertheless, differences in the mitochondrial membrane potential were not so remarkable, as monitored using rhodamine 123. When analyzing the same samples with Western blotting (Figure 7D), a reduction and a rise in LP-Slt2 levels were clearly observed in *tpk3∆* and *bcy1∆* mutants, respectively, confirming the relationship between the PKA pathway, oxidative stress, and the appearance of LP-Slt2 in the presence of CLT. Performing drop growth assays, we found that deletion of *TPK3* resulted in increased sensitivity to CLT but not to other stimuli of the pathway such as CR, which supports the specific implication of this PKA subunit in CLT-induced CWI pathway signaling (Figure 7E). Conversely, cells lacking Bcy1 were sensitive to either CLT or CR, which suggested that the lack of this protein can impair cell growth in any stress condition.

### 3.8. Conservation of Azole-Induced MAPK Signaling in Saccharomycotina

**Figure 8:**
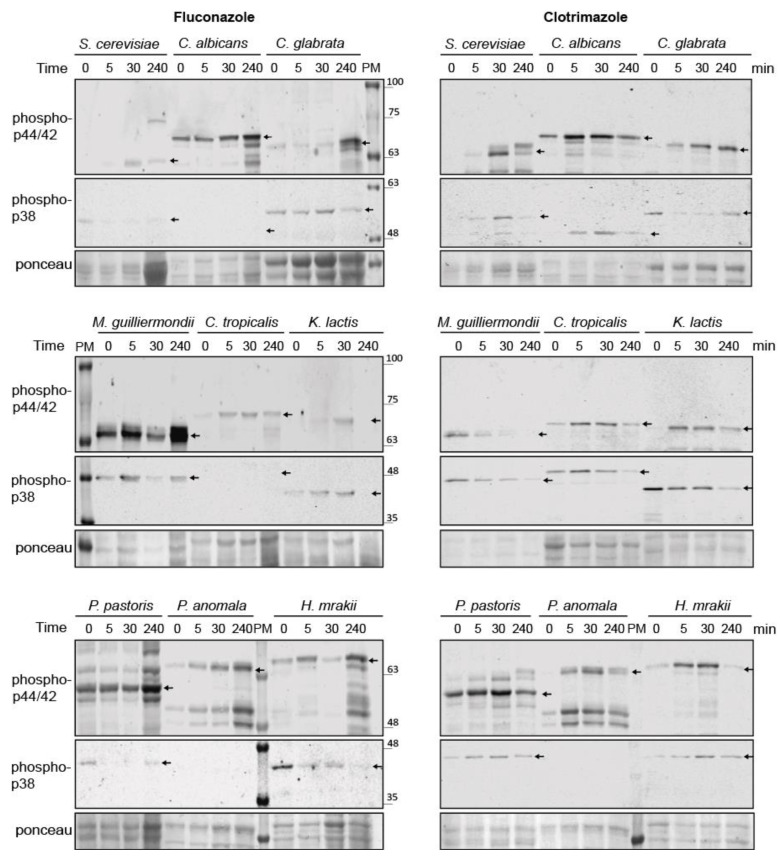
Activation of CWI and HOG pathways by azoles in other yeast species. Western blotting analysis of extracts of *Saccharomyces cerevisiae* BY4741, *Candida albicans* 4482A, *Candida glabrata* CBS138, *Meyerozyma guilliermondii* TP11010, *Candida tropicalis* 4458, *Kluyveromyces lactis* 1049, *Pichia pastoris* GS115, *Pichia anomala* 1026, and *Hansenula mrakii* K9 strains. Cells were cultured with 200 µg/mL of fluconazole (left panels) or with 50 µg/mL of clotrimazole (right panels) for the indicated times at 30 °C. Dually phosphorylated MAPK homologues of Slt2 and Hog1 were detected with anti-phospho-p44/42 and anti-phospho-p38, respectively, and the corresponding bands are indicated by black arrows in the blots. Ponceau staining was used for the loading control. PM corresponds to the lane loaded with the molecular weight protein marker. Representative blots from three independent experiments are shown.

To investigate whether the MAPK-mediated response to azoles observed in *S. cerevisiae* is conserved in other yeast species, we compared the MAPK phosphorylation of distinct yeast species belonging to the Saccharomycotina subphylum (budding yeasts) in response to CLT and FLC. Wild-type strains of *Saccharomyces cerevisiae*, *Candida glabrata*, *Meyerozyma guilliermondii*, *Candida albicans*, *Candida tropicalis*, *Pichia anomala*, *Kluyveromyces lactis*, *Pichia pastoris*, and *Hansenula mrakii* species were grown in rich medium and treated with FLC or CLT, and dual phosphorylation of the corresponding Slt2 and Hog1 orthologues was analyzed at distinct time points by Western blotting using anti-phospho-p44/42 and anti-phospho-p38 antibodies, respectively, as these antibodies can recognize dually phosphorylated MAPKs at the activation loop from different organisms. As shown in Figure 8, the phosphorylated forms of Slt2 orthologues were detected with the anti-phospho-p44/42 antibody in all the yeast species, as the protein bands matched the predicted molecular weights of the corresponding MAPKs. In general, CLT induced a phosphorylation of the CWI pathway MAPK, which increased with time, with an activation peak at 30 min in most species. The exception was the phosphorylated MAPK from *M. guilliermondii*, whose levels decreased after CLT treatment. In the case of FLC, Slt2 orthologues were also phosphorylated, but the highest phosphorylation was generally reached at the longest time point of 240 min.

The anti-phospho-p38 antibody recognized all Hog1 orthologues excepting for *P. anomala*, whose extracts showed no signal against this antibody. As with Slt2 orthologues, CLT induced the highest level of HOG pathway phosphorylation at short time points (5 or 30 min), with the signal decreasing at the longest time point of 240 min. However, in the cases of *C. glabrata* and *M. guilliermondii*, MAPK phosphorylation levels decreased after CLT treatment. In contrast to CLT, the response to FLC through the HOG pathway was weak and heterogeneous between species. Despite a CLT-dependent Slt2 mobility shift seeming to be a feature of *S. cerevisiae* Slt2, activation of this MAPK upon azole treatment is conserved in other yeast species. Altogether, these results indicate that the CWI and HOG pathways respond to azoles in different budding yeasts. Since some of them are of clinical interest, the implication of these pathways in azole-resistance should be addressed.

## 4. Discussion

In many aspects, *S. cerevisiae* has guided our understanding of the eukaryotic cell for decades, and especially when studying fungal biology. Although it lacks pathogenicity, this yeast species is considered a useful model system for studying azole susceptibility, as it provides information on sterol metabolism and cellular signaling [23]. Surprisingly, although they are the most widely used antifungals, not much is known about the action of azoles on MAPK signaling in fungi. To gain more insight into the mode of action of azoles and, in particular, to discover how imidazole antifungals act on fungal MAPK pathways, we explored the effect of CLT on *S. cerevisiae* MAPK circuitries. Our results show that unlike triazoles, imidazoles trigger profound changes in MAPK signaling, affecting all four MAPKs operating in vegetative yeast cells. Whereas MAPKs Hog1 and Slt2 are activated, signaling through mating MAPKs Fus3 and Kss1 is significantly reduced in a Hog1-dependent manner following CLT treatment, suggesting that similar mechanisms known to downregulate the mating pathway by hyperosmotic stress [43] are likely operating under CLT treatment. However, the actual crosstalk mechanisms that regulate mutual modulation between these two pathways are still poorly understood. Among them, Hog1-dependent Ste50 phosphorylation was found to negatively regulate signaling toward Kss1 and Fus3 [43,59]. Since CLT activates the Sln1 branch of the HOG pathway and Ste50 operates both in the alternative Sho1 branch and in the mating pathway, this Ste50 negative feedback phosphorylation may be a mechanism prioritizing the most necessary among pathways sharing components against a condition that threatens survival. The sensitivity of mutants to CLT with disrupted signaling through the HOG or CWI pathways revealed the importance of prioritization of these pathways specialized in responding to stress conditions.

Knowledge is limited of the mode of activation of the HOG pathway by other stressors distinct from hyper-osmotic shock. As mentioned above, CLT activates the HOG pathway through stimulation of the Sln1 branch of the pathway. Other stimuli use this branch for activating Hog1, such as citric acid [60] or acetic acid [61]. Interestingly, sphingolipid or ergosterol depletion also triggers this pathway [62], and the alteration in the membrane fluidity during hypoxia, probably through ergosterol deficiency, also leads to Hog1 activation through the Sln1 branch [63]. Therefore, the depletion of ergosterol generated by CLT may account for the observed Hog1 activation. However, the treatment of yeast cells with the triazoles FLC and ITC did not result in Hog1 phosphorylation, suggesting that other biological effects are also necessary for triggering the HOG pathway. Here, we showed that oxidative stress underlies CLT-induced Hog1 phosphorylation. Accordingly, FLC was reported to lack significant ROS-inducing capacity in yeast [64], and compounds that generate oxidative stress, such as hydrogen peroxide [65], also signal to Hog1 through the Sln1 branch.

The activation of Hog1 is important for the subsequent activation of the CWI pathway in response to some stresses. This is the case with SDS [29] and zymolyase [42] treatments. Zymolyase, a mixture of cell-wall-digesting enzymes, promotes Slt2 activation partly because of Hog1-driven glycerol accumulation [66]. However, genome-wide expression studies found no induction of genes coding the prototypical glycerol-producing proteins *GPD1* and *GPD2* after treatment with antifungals targeting ergosterol biosynthesis, including clotrimazole [67]. Consistently, we found that Slt2 activation in response to CLT does not rely on previous Hog1 activation, suggesting independence and limited crosstalk between the HOG and CWI pathways under treatment with imidazoles. The most remarkable traits in imidazole-induced MAPK signaling are related to the CWI pathway. Treatment of yeast cells with either clotrimazole or ketoconazole promoted Slt2 MAPK phosphorylation not only at the TEY activation loop but also at additional sites, producing an Slt2 form of low electrophoretic mobility (LP-Slt2). Although phosphorylation sites other than T190 and Y192 were reported [13], no changes in mobility such as that induced by CLT have been observed to date for Slt2. The strong Slt2-dependent phosphorylation of Rlm1 and Swi6 suggests that LP-Slt2 is fully catalytically active, at least on Rlm1 and Swi6. Therefore, the extra phosphorylation on Slt2 does not seem to negatively impact the activity of MAPK Slt2. Although the effects on the kinase activity and functionality merit further research, this additional post-translational modification is likely to impose an additional layer of regulation on Slt2.

Another striking feature of CLT-induced signaling is Pkc1-independent Slt2 activation. To the best of our knowledge, this is the first stimulus of the CWI pathway for which Pkc1 is not fully required for Slt2 activation, indicating that a novel signaling circuitry is operating under this stress. Under this condition, none of the Rho1 GEFs are necessary for signaling through the route, which also suggests the formation of alternative protein complexes. Nevertheless, the mechanosensor Wsc1 seems to be at least partially implicated in CLT sensing and transduction of the signal. We unsuccessfully attempted to find an alternative protein kinase distinct to Pkc1 that is able to activate the Slt2 MAPK module. Therefore, although the most presumable possibility is a surrogate kinase operating in this non-canonical Pkc1-independent signaling, other phosphorylation-independent mechanisms that can enhance signaling through the MAPK module cannot be ruled out. This is another example of the extraordinary plasticity of MAPK pathways, as recently observed with RAF-independent ERK phosphorylation in mammalian cells [68]. Although dispensable for Slt2 activation, we showed that Pkc1 is essential for cell viability under CLT stress. This finding is in line with the increased efficacy of antifungal drugs targeting the cell membrane, such as fluconazole, following the pharmacological inhibition of PKC [22]. These authors suggested that PKC1 enables tolerance to drugs that affect the cell membrane via the MAPK cascade, because the lack of MAPK module components results in sensitivity to these compounds [22]. However, in contrast to the absence of Slt2, we showed that the lack of Pkc1 promotes a non-osmotically remediable CLT sensitivity, indicating additional functions to the activation of the MAPK cascade for Pkc1 in this condition. Several targets, in addition to the Slt2 MAPK cascade, were already found for Pkc1, as well as roles for this kinase independent of CWI signaling [69] that can account for this phenotype.

We suggest that imidazole-induced CWI pathway rewiring occurs due to a combination of membrane alteration and intense oxidant activity. Different evidence supports this model. First, both FLC and CLT are antifungal agents that target Erg11, affecting the ergosterol biosynthesis pathway, as readily observed by their similar chemical–genetic profiles [70]. However, whereas FLC induces only a limited amount of ROS [64], here, we showed that CLT, which promotes LP-Slt2 formation, generates a high level of ROS in yeast, similar to the strong oxidant hydrogen peroxide (H_2_O_2_). Second, the antioxidant N-acetylcysteine eliminates the Slt2 band shift and significantly reduces Slt2 phosphorylation in response to CLT, indicating the importance of oxidative stress in CWI pathway activation in response to this imidazole. Notably, oxidative stress generated by other compounds, such as hydrogen peroxide or diamide, leads to Slt2 phosphorylation, but does not trigger the phosphorylation pattern observed for CLT (our unpublished results, [71]). Third, CPZ, one of the most-used antipsychotic and neuroleptic drugs, damages cell membranes and affects transport activity. CPZ also induces the unfolded protein response and inhibits protein synthesis [72]. Interestingly, CPZ also induces oxidative stress with high ROS generation not only in mammalian [73] but also in yeast cells [51], in which it also promotes an Slt2 phosphorylation pattern similar to CLT, suggesting the involvement of the same MAPK signaling circuitry. Fourth, the activity of the PKA pathway was linked to mitochondrial damage and ROS generation via Tpk3, which is a master regulator of mitochondrial activity. Cells lacking this catalytic subunit of the PKA show decreased respiratory activity and mitochondrial content [58]. Actin aggregation induces Ras/cAMP/PKA signaling, leading to the production of ROS from dysfunctional mitochondria [53,55]. Under this condition, the deletion of *TPK3* is sufficient to prevent the production of ROS [55]. Here, we showed that Slt2 phosphorylation correlates with PKA activation in response to CLT, with the PKA catalytic subunit Tpk3 being important for LP-Slt2 formation. Together, it is thus likely that the actin aggregation promoted by CLT can contribute to PKA-mediated mitochondrial alteration and ROS production, leading to CWI pathway activation. In this regard, distinct components of the CWI pathway, including Bck1, Mkk1, and Slt2, were found to localize to mitochondria under oxidative stress [74]. Moreover, Slt2 is required for mitophagy [75]. Therefore, it is tempting to speculate that the CWI pathway may sense oxidative stress at the mitochondria, and thus be involved in their mitophagy. In this line, it was observed that fungicidal drugs induce the switch from fermentative growth to ROS-producing mitochondrial respiration [56], and mitophagy is usually induced by prolonged respiration [76].

In addition to *S. cerevisiae*, the sub-phylum Saccharomycotina contains over 1000 other known species of budding yeasts, with more than 300 with a known genome, including the opportunistic pathogens *Candida albicans* and *Candida glabrata* [77]. Conservation occurs in many aspects between *S. cerevisiae* and pathogen yeasts, including some canonical drug resistance mechanisms [78]. Our studies with different budding yeasts of this sub-phylum highlight the evolutionary conservation of the CWI and HOG pathways as stress-responsive pathways following azole treatment. Defining the differences and similarities between *S. cerevisiae* and pathogenic fungi underpinning the response to azoles will enable the rational design of combination therapies to tackle resistance to these antifungal drugs.

## Data Availability

The data presented in this study are available on request from the corresponding authors.

## Supplementary Materials

The following are available online at https://www.mdpi.com/article/10.3390/jof7080647/s1, Figure S1: Analysis of the role of Pkc1 in clotrimazole signaling through the CWI pathway in the CML128 background. Figure S2: Effect of clotrimazole on cells bearing the Integrity Pathway Activation Circuit (IPAC).
