# Supplementary material for: Clotrimazole-Induced Oxidative Stress Triggers Novel Yeast Pkc1-Independent Cell Wall Integrity MAPK Pathway Circuitry
Source: J Fungi (Basel). 2021 Aug 9;7(8):647. doi: 10.3390/jof7080647 (PMC8399625; doi:10.3390/jof7080647)
Supplement: Supplementary file 1 [file jof-07-00647-s001.zip › jof-1318680-supplementary.pdf]

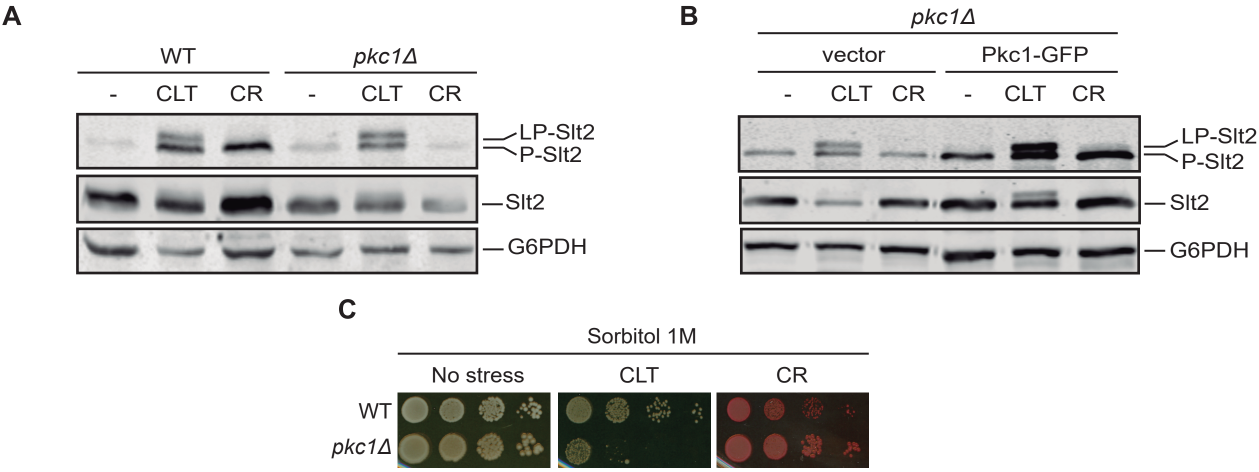

Figure S1. Analysis of the role of Pkc1 in clotrimazole-signaling through the CWI pathway in the CML128 background. (A) Western blotting analysis of extracts of CML128 (WT) and its isogenic *pkc1Δ* strain. Cells were incubated without stimulus (-), with 50 µg/mL of CLT, or 30 µg/mL of CR for 4 h at 24 ºC. Dually phosphorylated Slt2, Slt2 protein, and G6PDH (as the loading control) were detected with anti-phospho-p44/42, anti-Mpk1, and anti-G6PDH, respectively. (B) Western blotting analysis of *pkc1Δ* cells transformed with the empty vector pRS316 or with pVD67 (Pkc1-GFP). Cells were cultured and proteins were detected as in (A). Representative blots from three (A) and two (B) independent experiments are shown. (C) Sensitivity of the same strains as in (A) determined according to a drop dilution growth assay. Ten-fold serial dilutions of cell suspensions were spotted onto YPD plates supplemented with 1 M sorbitol in the absence (no stress) or presence of 1 µg/mL of CLT or 30 µg/mL of CR and incubated at 30 ºC for 72 h. A representative assay from two independent experiments is shown.

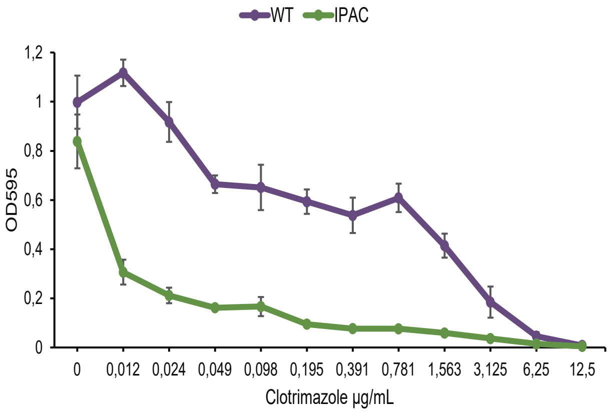

Figure S2. Effect of clotrimazole on cells bearing the Integrity Pathway Activation Circuit (IPAC). Multi-well-plate sensitivity assay determining sensitivity of the YSTH1 (WT) and YSTH2 (IPAC) strains to the indicated concentrations of clotrimazole. Cells were incubated at 30 ºC for 24 h, and optical density was measured at 595 nm (OD595). Data are presented as the average value of three independent experiments. Error bars indicate the standard deviation.
